# Myelodysplastic syndrome in a patient with HCV cirrhosis. Case report

**DOI:** 10.1186/1471-2334-13-S1-P94

**Published:** 2013-12-16

**Authors:** Mihai Olariu, Florin Căruntu, Cristina Olariu, Camelia Dobrea, Otilia Georgescu

**Affiliations:** 1 National Institute for Infectious Diseases “Prof. Dr. Matei Balş”, Bucharest, Romania; 2 Carol Davila University of Medicine and Pharmacy, Bucharest, Romania; 3 Fundeni Clinical Institute, Bucharest, Romania

## Background

Myelodysplastic syndromes are clonal myeloid disorders marked by ineffective hematopoiesis, cytopenia and qualitative disorders of blood cells that have a variable predilection to undergo clonal evolution to acute myelogenous leukemia. Chronic infection with hepatitis C virus (HCV) induces a chronic stimulation of B lymphocytes and, in some cases, this stimulation can lead to chronic lymphoid disorders like non-Hodgkin lymphoma.

## Case report

We report a case of myelodysplastic syndrome (overt multilineage dysmorphic cytopenia) in a 54 year-old man who had HCV cirrhosis with HCV. The patient was diagnosed with cirrhosis in December 2011 in our Institute. From 2011 to April 2013 the blood counts were relatively normal with mild to moderate thrombocytopenia. On April 2013 the patient presented with pallor, asthenia, weakness and dyspnea. Blood count showed pancytopenia with severe anemia: hemoglobin 6.2 g/dL. We performed a bone marrow biopsy that indicated the morphological changes on erythropoiesis, granulopoiesis and thrombopoiesis. The patient received red cells transfusion and he was transferred to the Fundeni Clinical Institute where cytogenetic abnormalities showed loss of Y chromosome in 4 metaphases. The patient received recombinant human erythropoietin every month and the blood count in June 2013 was as follows white blood cells 3.4 x 10^9^/L, hemoglobin 13 g/dL, platelets 85 x 10^9^/L.

## Conclusion

In the past few years we have observed a number of cases with HCV infection that associated myelodysplastic syndromes and our question is if HCV may be implicated in the etiology of these syndromes, but further studies are necessary to verify this hypothesis.

